# Ethacridine inhibits SARS-CoV-2 by inactivating viral particles

**DOI:** 10.1371/journal.ppat.1009898

**Published:** 2021-09-03

**Authors:** Xiaoquan Li, Peter V. Lidsky, Yinghong Xiao, Chien-Ting Wu, Miguel Garcia-Knight, Junjiao Yang, Tsuguhisa Nakayama, Jayakar V. Nayak, Peter K. Jackson, Raul Andino, Xiaokun Shu

**Affiliations:** 1 Department of Pharmaceutical Chemistry, University of California, San Francisco, San Francisco, California, United States of America; 2 Cardiovascular Research Institute, University of California, San Francisco, San Francisco, California, United States of America; 3 Department of Microbiology and Immunology, University of California, San Francisco, San Francisco, California, United States of America; 4 Department of Baxter Laboratory for Stem Cell Biology, Department of Microbiology & Immunology, Stanford University, California, United States of America; 5 Department of Otolaryngology–Head and Neck Surgery, Stanford University, Stanford, California, United States of America; The Peter Doherty Institute and Melbourne University, AUSTRALIA

## Abstract

The respiratory disease COVID-19 is caused by the coronavirus SARS-CoV-2. Here we report the discovery of ethacridine as a potent drug against SARS-CoV-2 (EC_50_ ~ 0.08 *μ*M). Ethacridine was identified via high-throughput screening of an FDA-approved drug library in living cells using a fluorescence assay. Plaque assays, RT-PCR and immunofluorescence imaging at various stages of viral infection demonstrate that the main mode of action of ethacridine is through inactivation of viral particles, preventing their binding to the host cells. Consistently, ethacridine is effective in various cell types, including primary human nasal epithelial cells that are cultured in an air-liquid interface. Taken together, our work identifies a promising, potent, and new use of the old drug via a distinct mode of action for inhibiting SARS-CoV-2.

## Author summary

To identify inhibitors against SARS-CoV-2, here we have designed and demonstrated a high throughput screening system that works in living cells at BSL2. To repurpose existing drugs, we have screened an FDA-approved drug library and identified several drugs that inhibit Mpro and block SARS-CoV-2 in cells. Interestingly, one of the identified drugs, ethacridine, shows ~40-fold higher antiviral activity than its antiprotease activity. Further studies unveil that ethacridine inhibits the coronavirus by inactivating the viral particles. Our work demonstrated a powerful tool to screen protease inhibitors to inhibit coronavirus, as well as other diseases in the future.

## Introduction

The worldwide outbreak of the respiratory disease COVID-19 is caused by the coronavirus SARS-CoV-2 (severe acute respiratory syndrome coronavirus 2). SARS-CoV-2 is an RNA betacoronavirus of the family *Coronaviridae*. It contains a single-stranded positive-sense RNA genome encapsulated within a membrane envelope [1–4]. Its genomic RNA is approximately 30kb with a 5′-cap structure and 3′-poly-A tail [1]. The genome of SARS-CoV-2 can be split into two main regions that contain as many as 14 open reading frames (ORFs) [5].

The first region, containing the first ORF (ORF1a/b), is about two-thirds of the genome. After coronavirus attach and entry into the host cell, the viral genomic RNA is released. The first region of the genomic RNA is translated to pp1a and pp1ab. The pp1a polyprotein is translated from ORF1a and the pp1ab polyprotein comes from a -1 ribosomal frameshift between ORF1a and ORF1b. Both pp1a and pp1ab are mainly processed by a 3-chymotrypsin-like protease (3CLpro, referred as the main protease, Mpro). Mpro cleaves the polyproteins in at least 11 conserved sites. The functional polypeptides including 16 non-structural proteins (nsp1–16) are released from the polyproteins after this extensive proteolytic processing. Among them, Nsp12 (i.e., RNA-dependent RNA polymerase (RdRp)), together with other nsps (e.g., nsp7 and nsp8), forms a multi-subunit replicase/transcriptase complex (RTC) that is associated with the formation of virus-induced double-membrane vesicles [4,6,7]. The membrane-bound RTC synthesizes a full-length negative-strand RNA template for the positive-strand viral genomic RNA.

The remaining one-third of the genomic RNA is used by the RTC to synthesize subgenomic RNAs (sgRNAs) encoding four conserved structural proteins including spike protein (S), envelope protein (E), membrane protein (M), and nucleocapsid protein (N), and several accessory proteins. Eventually, the viral RNA-N complex and S, M, and E proteins are assembled in the ER-Golgi intermediate compartment (ERGIC) to form a mature virion that is then released via budding from the host cell. S protein is exposed on the surface of the virion and binds human angiotensin converting enzyme 2 (ACE2) on the host cell surface. Therefore, Mpro plays a central and critical role in the lifecycle of the coronavirus and is an attractive drug target [8–10], which also include other biological steps essential for viral replication and budding.

To identify drugs that may inhibit the coronavirus, we redesigned the green fluorescent protein (GFP) into an activity reporter, which becomes fluorescent only upon cleavage by the active Mpro. Using this fluorescent assay, we screened a drug library in living cells and identified several drugs that inhibit Mpro activity. One highly effective drug, ethacridine, inhibit SARS-CoV-2 production by inactivating viral particles.

## Results

### Rational design of a fluorogenic Mpro activity reporter FlipGFP^Mpro^

**Fig 1:**
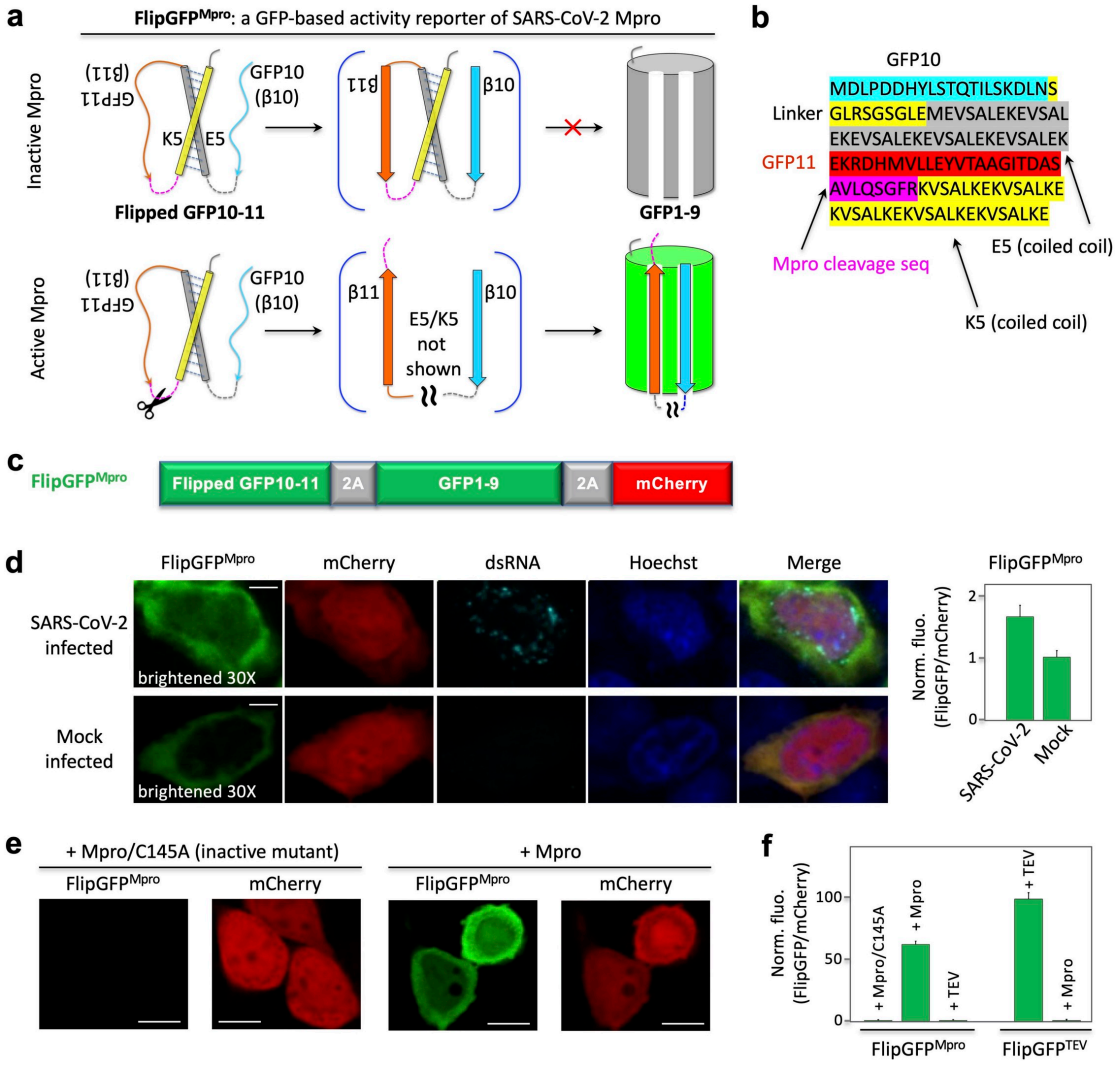
Design and demonstration of a GFP-based activity reporter of SARS-CoV-2 main protease Mpro. **(a**) Schematic of the reporter. (**b**) Sequence of the flipped GFP10-11. (**c**) Construct of the reporter FlipGFPMpro. (**d**) Fluorescence images (left) and quantitative analysis (right) of SARS-CoV-2 or mock-infected HEK293T cells that co-expressed hACE2. The images in the FlipGFP channel were brightened 30-fold compared to those in (**e**). (**e**) Fluorescence images of HEK293T cells expressing FlipGFPMpro and mCherry, together with the inactive Mpro mutant C145A (upper panels) or wild type Mpro (lower panels). (**f**) Normalized FlipGFP fluorescence by mCherry. The ratio of FlipGFP/mCherry for the Mpro/C145A is normalized to 1. Data are mean ± SD (n = 5). FlipGFPTEV is a TEV activity reporter containing TEV cleavage sequence in FlipGFP. Scale bar: 5 μm (**d**); 10 μm (**f**).

To develop an activity reporter of Mpro with a large dynamic range suitable for high-throughput screening (HTS), we applied the GFP-based protease reporter called FlipGFP [11], which was designed by flipping one of the 11 beta-strands of a split GFP. Briefly, the split GFP contains two parts: one part contains beta-strands 10 and 11 (i.e., GFP10 and 11), and the other contains nine other beta-strands and the central alpha helix (i.e., GFP1–9). GFP10–11 contains the highly conserved Glu222 that is essential for catalyzing chromophore maturation. GFP1-9 contains the three amino acids that form the chromophore via cyclization, dehydration and oxidation [12]. GFP10-11 spontaneously binds GFP1-9 and catalyzes the chromophore maturation, leading to green fluorescence. To design an Mpro activity reporter, we “flipped” GFP10-11 using heterodimeric coiled coils (E5 and K5) so that the flipped GFP10-11 cannot bind GFP1-9 when Mpro is inactive, and thus, no or little fluorescence is detected (**Fig 1A**). We incorporated an Mpro-specific cleavage sequence AVLQ↓SGFR (↓denotes the cleavage site) between GFP11 and K5. In this way, when Mpro cleaves the proteolytic site, GFP11 is flipped back, allowing GFP10-11 to bind GFP1-9, resulting in bright fluorescence (**Fig 1A**). We named this reporter FlipGFP^Mpro^. To normalize the fluorescence, we added a red fluorescent protein mCherry within the construct via a “self-cleaving” 2A peptide [13] (**Fig 1C**).

To determine if FlipGFP^Mpro^ serves to report on Mpro activity of SARS-CoV-2 in living cells, we expressed ACE2, a SARS-CoV2 receptor, in HEK293-FlipGFP^Mpro^ cells. Next, we infected the cells with SARS-CoV-2, and at 24 hours post-infection, cells were analyzed by immunofluorescence using antibodies against double-stranded RNA (dsRNA) and FlipGFP^Mpro^ green fluorescence. The green fluorescence of the sensor, normalized by the co-expressed mCherry, was 63% greater in the coronavirus-infected cells than in mock-infected cells (**Fig 1D**). Infected cells also showed dsRNA fluorescence compared to non-infected (mock) cells without dsRNA staining (**Fig 1D**). These data demonstrate that the utility of FlipGFP^Mpro^ as a reporter of SARS-CoV-2 Mpro activity in human cells.

Next, we established a system for screening Mpro inhibitors in living cells by exogenously expressing Mpro in HEK293 cells. Specifically, wild-type Mpro or an inactive Mpro mutant (with catalytic cysteine 145 mutated to alanine) were co-expressed in this cell line. The green fluorescence of FlipGFP^Mpro^ was barely detected in the cells expressing the inactive Mpro/C145A mutant, whereas the red fluorescence of mCherry was observed (**Fig 1E**, upper panels). On the other hand, strong green fluorescence was detected in the cells expressing Mpro with similar levels of mCherry fluorescence (**Fig 1E**, lower panels). The green fluorescence of FlipGFP^Mpro^, normalized to the red fluorescence of mCherry, revealed an ~60-fold dynamic range between inactive and active Mpro (**Fig 1F**). Furthermore, based on these quantified data, we calculated a *Z*’*-factor* [14]

$$Z=1-\frac{\left(3\sigma_{+}+3\sigma_{-}\right)}{\left|\mu_{+}{-\mu }_{-}\right|},$$

which is ~0.8 with Mpro and its inactive mutant as positive (+) and negative (-) controls, respectively (here *σ* is standard deviation, *μ* is mean). This suggests that the assay is robust for HTS.

The FlipGFP^Mpro^ sensor was not responsive to the TEV protease, and the FlipGFP-based TEV reporter (FlipGFP^TEV^) was only activated by TEV but not by Mpro (**Fig 1F**). Thus, FlipGFP^Mpro^ specifically detects Mpro activity with a large dynamic range. Therefore, our data show that we have established a robust HTS system for screening Mpro inhibitors at a BSL2 level with 60-fold fluorescence change and a robust z’-factor. Difference of the normalized FlipGFP^Mpro^ fluorescence in the SARS-CoV-2 infected cells (**Fig 1D**) and that in the cells expressing Mpro exogenously (**Fig 1E** and **1F**) suggests that the active Mpro concentration in the cytoplasm of the coronavirus-infected cells is ~100-fold lower than that of the HEK293 cells exogenously expressing Mpro (under a EF1*α* promoter).

### HTS of drugs that inhibit Mpro activity in living cells

**Fig 2:**
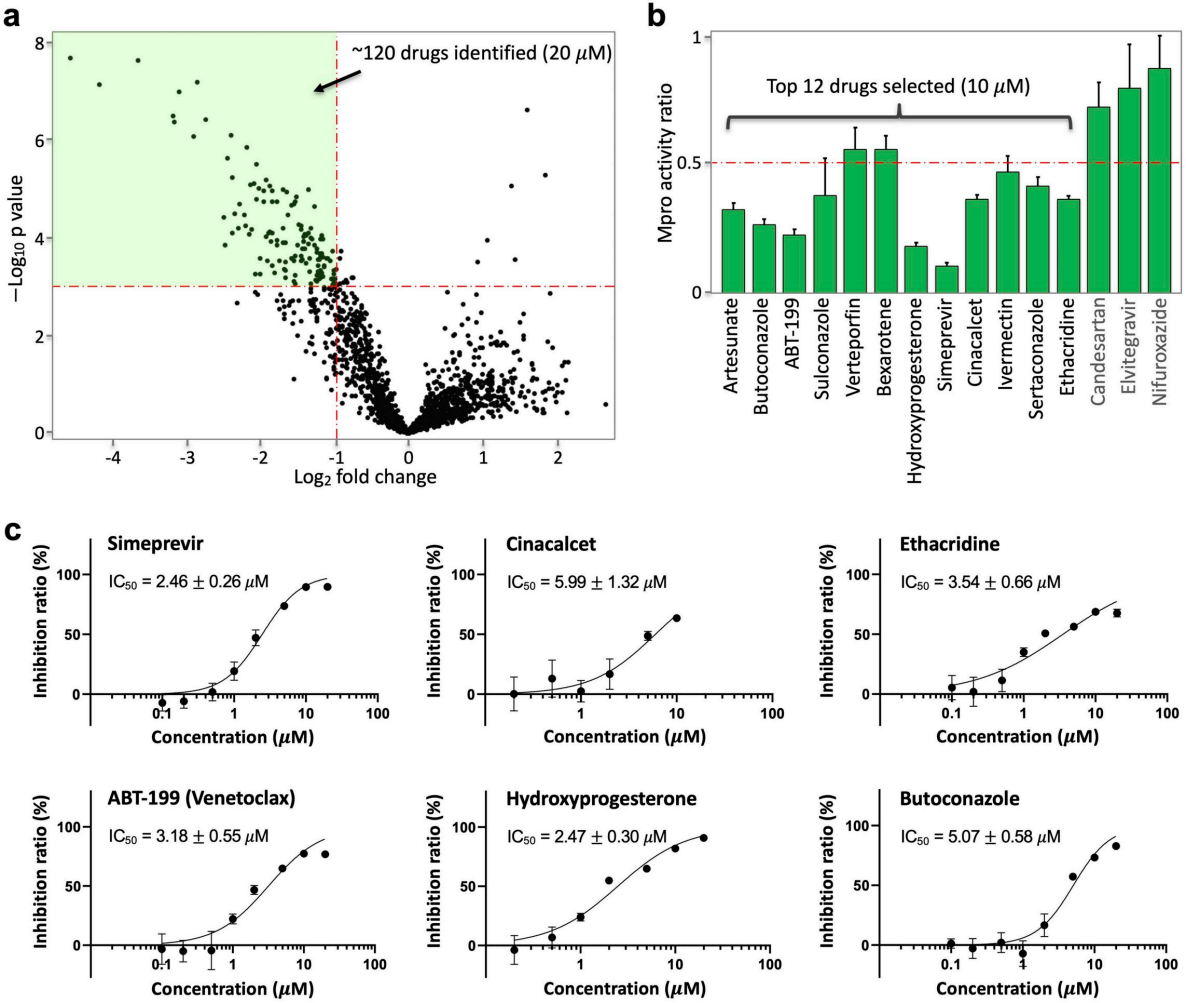
High-throughput screening and drug identification using FlipGFPMpro in living cells. (**a**) Volcano plot of 1622 FDA-approved drugs (20 μM) in inhibiting Mpro. HEK293T cells were transfected with FlipGFPMpro, mCherry and Mpro. FlipGFP fluorescence was normalized to co-expressed mCherry. (**b**) Normalized ratio of Mpro activity in drug (10 μM) vs DMSO-incubated cells for the third-round validation. The Mpro activity was determined as FlipGFP fluorescence normalized to mCherry. The ratio of Mpro activity was calculated by normalizing Mpro activity with that of cells treated with DMSO. Data are mean ± SD (n = 5). The 15 drugs were identified from a second-round imaging of the 120 identified drugs (20 μM, Extended data Fig 1). (c) Dose-response curve of top six drugs in inhibiting Mpro. Inhibition ratio was calculated as (1-(ratio of Mpro activity)) X100%. IC50 was represented as mean ± SEM (n = 5). See Extended data Fig 2 for the other six drugs.

Next, we conducted HTS of ~1600 FDA-approved drugs (20 *μ*M final concentration, **Fig 2A**). The reporter construct (FlipGFP^Mpro^ and mCherry) was transfected into HEK293 cells, followed by addition and incubation of the drugs. Green fluorescence normalized to red fluorescence were then calculated. A volcano plot revealed ~120 drugs that showed ≥ 50% reduction of Mpro activity with a p-value < 0.001 (**Fig 2A**). To confirm this result, we re-screened the identified ~120 drugs under similar conditions (**S1 Fig**). We further assayed those top 15 drugs at a lower concentration (10 *μ*M) and found that 12 drugs showed ≥50% reduction of FlipGFP^Mpro^ fluorescence (normalized by mCherry) at 10 *μ*M concentration (**Fig 2B**). We finally calculated an IC_50_ for each of the 12 drugs. IC_50_ of six drugs were at 2–6 *μ*M (**Fig 2C**), and the rest were above 6 *μ*M (**S2 Fig**). Lastly, we also determined cellular viability of these identified drugs in HEK293T cells, which showed that they are not toxic at the concentrations that inhibit Mpro activity (**S3 Fig**).

### Antiviral activity of identified drugs

**Fig 3:**
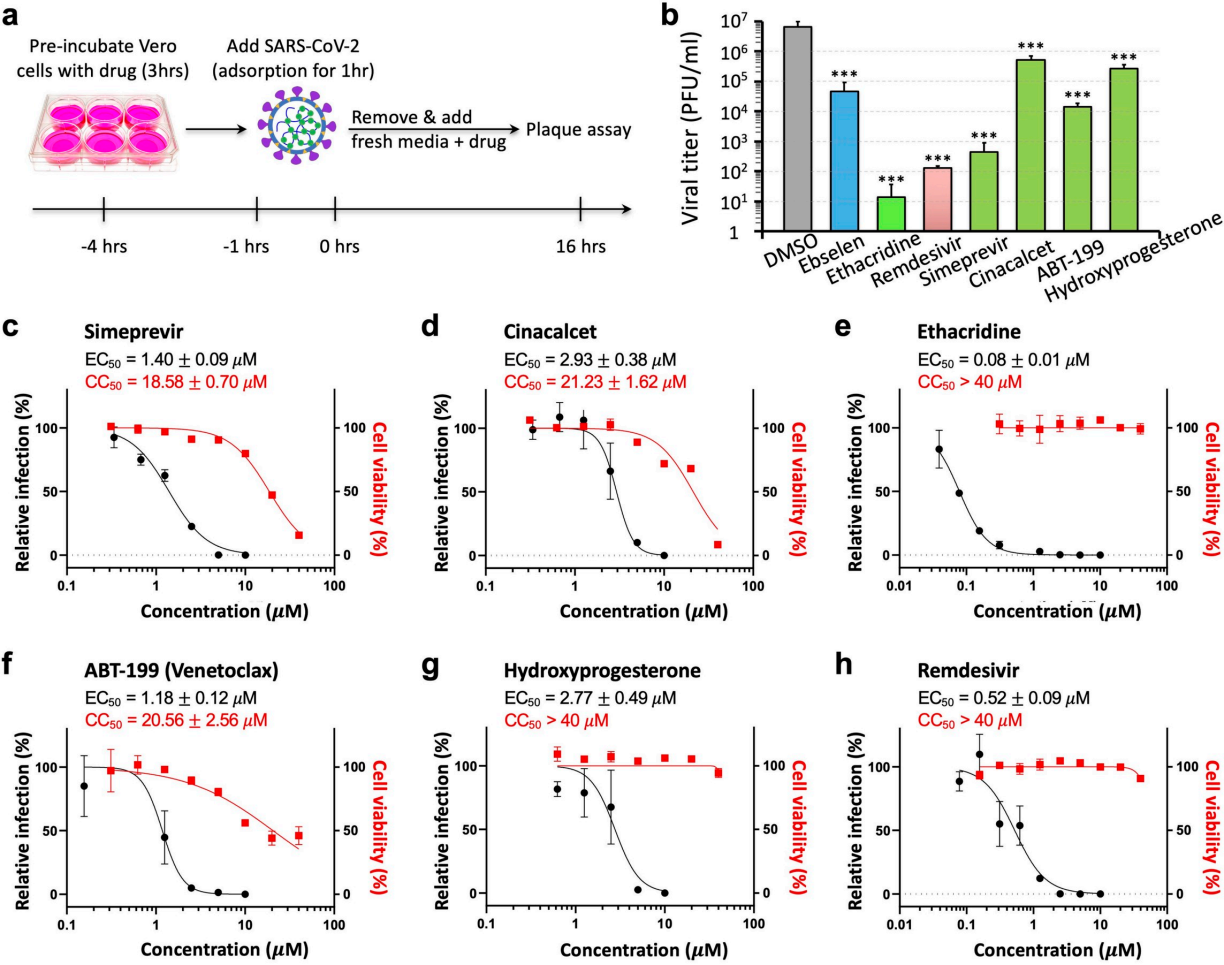
Antiviral activities of the identified drugs against SARS-CoV-2. (**a**) Schematic showing the experimental design, see Methods for details. (**b**) Quantitative analysis of viral titer from plaque assays on Vero E6 cells treated with each drug at 5 μM. Data are mean ± SEM (n = 3). *** *p* < 0.001. (**c–h**) Dose-response and cell-toxicity curve of each drug against SARS-CoV-2 by plaque assays. The percentage of relative infection was determined by the ratio of infection rate of SARS-CoV-2 treated with each drug divided by that of DMSO control. EC50 and CC50 are represented as mean ± SEM (n = 3).

We next investigated antiviral activity of selected drugs in Vero E6 cells. The cell monolayers were pretreated with the 12 selected drugs for 3 hours, and then infected with SARS-CoV-2. The cells were further cultivated in the presence of each respective compound at a concentration of 5 μM. After 16 hours of incubation, the culture media samples were collected, and the amount of infectious particles were estimated by plaque assay (**Figs 3A** and **3B** and **S4**). Our data revealed that 9 of the 12 drugs showed significant antiviral activity at 5 μM. Strong inhibition was detected for ethacridine with 5–6 logs reduction in viral titer, simeprevir ~4-log reduction, ABT-199 ~2-log reduction, hydroxyprogesterone ~1-log reduction, cinacalcet ~1-log reduction. Two of the 12 drugs (ivermectin and verteporfin) were cytotoxic at 5–13 μM in Vero E6 cells and excluded from further analysis (**S5 Fig**). As a comparison, we tested the antiviral effect of a reported Mpro inhibitor, ebselen, which showed ~2-log reduction in viral titer. The RdRp inhibitor remdesivir showed ~4-log reduction.

Next, we determined dose-response curves for the top 5 selected drugs. First, our data revealed that the EC_50_ of four drugs (simeprevir, cinacalcet, ABT-199 and hydroxyprogesterone) was 1–3 μM (**Fig 3C–3G**), within a range similar to their IC_50_ in inhibiting Mpro (**Fig 2C**). This was consistent with the expectation that SARS-CoV-2 replication is inhibited by restricting Mpro activity. Indeed, as we were finalizing this study, a preprint report showed that simeprevir inhibits Mpro activity and SARS-CoV-2 [15]. By contrast, ethacridine showed outstanding antiviral activity (EC_50_ ~ 0.08 μM, **Fig 3E**), which is 40-fold lower (i.e. stronger) than its Mpro-inhibiting activity (IC_50_ ~ 3.54 μM, **Fig 2C**). These data suggest that the antiviral activity of ethacridine is not mainly accounted for by its Mpro-inhibiting activity. Lastly, for comparison, we also determined the EC_50_ of remdesivir ~0.52 μM in a side-by-side manner (**Fig 3H**), which indicates that ethacridine is more potent than remdesivir.

### Ethacridine inhibits SARS-CoV-2 by inactivating viral particles

**Fig 4:**
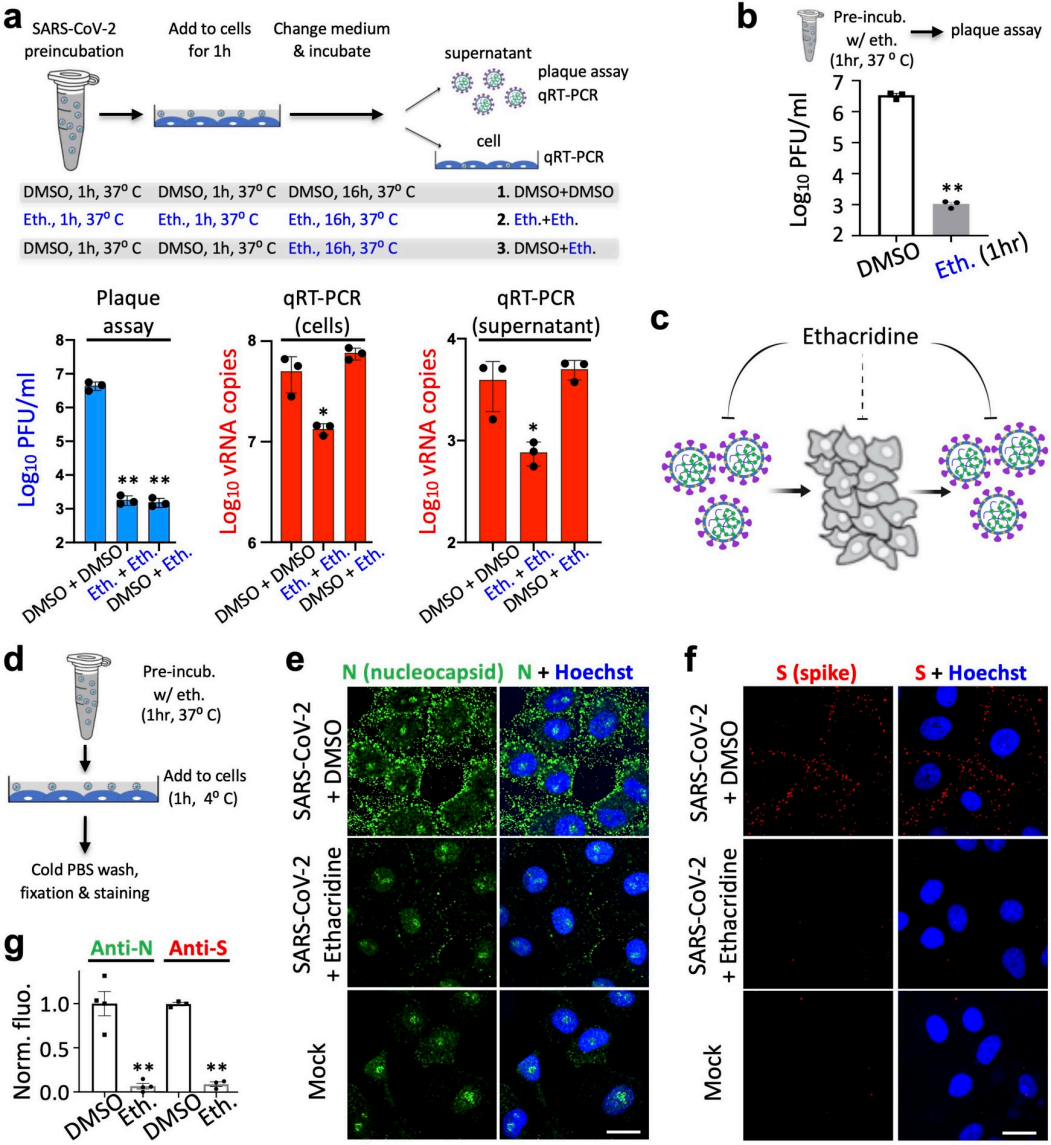
Ethacridine bocks SARS-CoV-2 by inactivating viral particles. (**a**) Upper panel: schematic showing the experimental design for plaque assay and qRT-PCR. The virus was pre-incubated with ethacridine or DMSO for 1 hr. The mixture was added to Vero cells for adsorption at 37°C for 1 hr. Details can be found in the text. Lower panel: quantitative analysis of viral titer from plaque assay (left), and viral RNA (vRNA) copies by qRT-PCR in Vero cells (middle) and supernatant (right). (**b**) Quantitative analysis of viral titer by plaque assay. (**c**) Proposed mode of action of ethacridine by mainly inactivating viral particles of the coronavirus with no or little effect on viral RNA replication. (**d**) Schematic showing the experimental design for immunostaining. (**e**, **f**) Representative images of immunostaining against nucleocapsid protein (N) and spike (S) in Vero E6 cells after infection with the virus that was pre-treated with ethacridine (5 μM) or DMSO (control), or no infection (mock). (**g**) Quantitative analysis of the immunofluorescence. Data are mean±SEM (n = 3 or 4 biological replicas). **: p value < 0.01. *: p value < 0.05. Scale bar, 20 μm.

To determine how ethacridine inhibits SARS-CoV-2, we tested infectivity of the virus particles after ethacridine treatment with plaque assay, and we also measured viral RNA levels using qRT-PCR. We examined the antiviral effect of ethacridine on different stages of the lifecycle of SARS-CoV-2, including virus-cell binding, RNA replication, and budding. To test overall effect of ethacridine on virus replication, we pre-incubated SARS-CoV-2 particles with ethacridine (5 μM) or DMSO control for 1 hour. The mixture was then added to Vero E6 cells for viral adsorption at a multiplicity of infection (MOI) at 0.5. Next, we removed the solution and added fresh medium containing ethacridine (5 μM) or DMSO control. Sixteen hours later, we collected supernatant and conducted plaque assay with overlaid agar without ethacridine or DMSO to measure viral titer. We also conducted qRT-PCR and measured viral RNA levels in the supernatant and within cells. In this way, we developed three conditions (**Fig 4A**): 1) Control (DMSO + DMSO): the virus and cells were exposed to DMSO and not the drug; 2) The virus and cells were exposed to the drug at all stages, including 1 hour before infection, during replication, and after viral budding (i.e. Eth. + Eth.); 3) The virus and cells were exposed to the drug only after viral entry, during replication, and after budding (i.e. DMSO + Eth.). Lastly, we used a fourth condition (**Fig 4B**): we conducted plaque assay right after pre-treatment of SARS-CoV-2 with ethacridine for 1 hr (i.e. Eth. [1 hr]), which determines direct effect of the drug on viral particles.

When ethacridine was present continuous prior to plaque assay (Eth. + Eth.), viral titers were reduced 3–4 logs, compared with the control (DMSO + DMSO) (**Fig 4A**, lower left panel). When ethacridine was added to the Vero cells after viral entry (DMSO + Eth.), similar level of reduced infectivity (3–4 logs reduction in viral titer) was observed (**Fig 4A**). Importantly, when SARS-CoV2 was pre-incubated with ethacridine for 1 hr (Eth. [1 hr]) and followed by plaque assay without drug, we also observed 3–4 logs reduction in infectivity (**Fig 4B**). This result suggests that the drug directly inactivates SARS-CoV2 viral particles. Because of similar-level reduction in infectivity in all of the three conditions, our data strongly suggests that ethacridine inhibits SARS-CoV-2 mainly by inactivating viral particles.

We next examined viral RNA accumulation in infected cells. qRT-PCR measurement revealed no change of viral RNA (vRNA) levels when the drug was added after viral binding and cell entry (DMSO + Eth.) in both the supernatant and the cells (**Fig 4A**, lower middle and right panels), compared with the control (DMSO + DMSO). This indicates that the drug has no effect on vRNA replication. As the infectivity of supernatant from “DMSO + Eth” treated samples showed 3–4 logs reduction in plaque assay (**Fig 4A**, lower left panel), these data suggests that ethacridine inhibits SARS-CoV-2 by inactivating the viral particles without effect on vRNA replication. This is consistent with the results of plaque assays for the supernatant samples with 3 different treatments in that showed 3~4 log reduction in infectivity (**Fig 4A**, lower left panel and **Fig 4B**) after virions in the supernatant were exposed to the drug before plaque assay.

Next, when ethacridine was present continuously (i.e. Eth. + Eth.), 4–5 fold reductions were observed in vRNA copies in the supernatant and within cells (**Fig 4A**, middle and right panels). Because plaque assay-based measurement of the same conditioned sample (Eth. + Eth.) showed 2400-fold reduction in viral titer, the effect of ethacridine on viral replication (4–5 fold reduction) is about 500-fold smaller than its effect on viral infectivity. This further supports the conclusion that ethacridine inhibits SARS-CoV-2 by inactivating the viral particles. The 4–5 fold reduction of vRNA copies is likely due to reduced viral copy numbers that may bind to the cells (see below), because here the additional step is that the virus were pre-incubated with the drug.

Thus, our plaque assay and qRT-PCR data suggests that ethacridine inhibits SARS-CoV-2 mainly by inactivating viral particles, including the virus before binding to cells and in the supernatant after budding from host cells, with no or little effect on vRNA replication (**Fig 4C**).

To further investigate the mechanism of ethacridine-based inactivation of the viral particles, we conducted immunofluorescence staining and imaging to determine whether the ethacridine-treated SARS-CoV-2 can bind to the cells. We treated SARS-CoV-2 with ethacridine (5 μM) or DMSO for 1 hour at 37°C. Then the virus was added to cells for adsorption (4°C, 1 hour) at a MOI = 100. Cells were then quickly washed and fixed with 4% PFA (**Fig 4D**). Immunostaining with antibodies against the nucleocapsid protein (N) of SARS-CoV-2 showed strong anti-N fluorescence on the plasma membrane of the cells infected with control virus, but little anti-N fluorescence in cells exposed to ethacridine treated virus (**Fig 4E** and **4G**). Immunostaining against the Spike protein (S) of SARS-CoV-2 showed the same results (**Fig 4F** and **4G**). Furthermore, to examine whether ethacridine blocks viral binding to cells by perturbing the cellular factors for viral binding such as cellular receptors, we pre-treated cells with ethacridine for 3 hours. This was followed by washing and drug removal, immediately prior to addition of SARS-CoV-2. The data showed that viral infection was not affected by these procedures (**S6 Fig**), suggesting that the main effect of the drug is not on the cells, but on the viral particles. These results indicate that ethacridine-treated SARS-CoV-2 cannot bind cells to initiate infection.

To further support our model, we conducted an additional experiment. We mixed ethacridine with SARS-CoV-2 and immediately added the drug/virus mixture to the Vero cells (i.e. no preincubation of the drug with the virus). After adsorption for 1 hour (37°C), we overlayed the cells with agar and media for plaque assay. Under this condition, first, the drug will be able to inhibit potential cellular factors since the drug is not removed after the adsorption step. Second, the drug is not preincubated with the virus, and thus according to our model, we expect much smaller effect of the drug on the virus than when the drug was preincubated with the virus. Indeed, we observed dramatically smaller effect of the drug: ~2.7-fold inhibition (**S7 Fig**) versus 3–4 logs inhibition when the drug was preincubated with the virus (**Figs 4B** and **S6**). These results further support our model that ethacridine inhibits SARS-CoV-2 by mainly inactivating the viral particles.

We also tested the dependency of the viral-inactivation effect of ethacridine on dose, incubation time and incubation temperature with a plaque assay. For the conditions tested, the viral-inactivation effect showed dose-dependency but was comparable to a 1- or 2-hour incubation at room temperature or at 37°C (**S8 Fig**).

### Further validation in human cells

**Fig 5:**
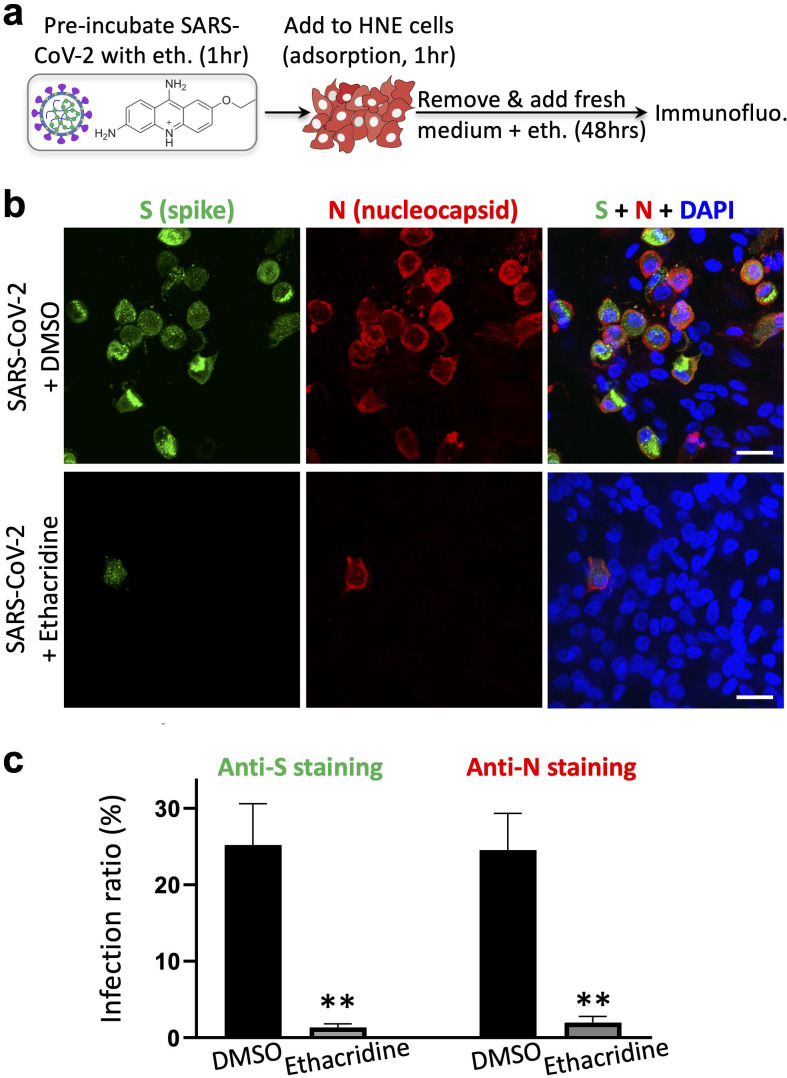
Ethacridine blocks SARS-CoV-2 in primary human nasal epithelial cells. (**a**) Schematic showing the experimental design, see Methods for details. (**b**) Representative images of immunostaining against spike (S) and nucleocapsid protein (N) in HNE cells 48 hours after viral infection in the presence ethacridine (5 μM) or DMSO (control), or no infection (mock). (**c**) Quantification of infection ratio based on anti-S or anti-N staining. Data are mean ± SEM (three biological replicates). **: p value < 0.01. Scale bar, 20 μm.

To ensure that the antiviral effect is not restricted to the Vero E6 cells, we have further evaluated the anti-viral effect of ethacridine in human cells. We used a human lung epithelial A549 cell line stably expressing human ACE2 (A549^ACE2^) and human primary nasal epithelial (HNE) cells. A plaque assay in A549^ACE2^ cells revealed that ethacridine-treated virus showed a dramatic decrease in infectivity when applied to A549^ACE2^ cells (**S9 Fig**), indicating that the effect of virus inactivation of ethacridine is not specific to Vero E6 cells. This is consistent with the suggested mode of action of ethacridine by inactivating SARS-CoV-2 particles. The cilia organelles within the nasal epithelium have been shown to strongly and specifically express the ACE2 receptor exploited by SARS-CoV-2 [16]. To validate the antiviral effects of ethacridine in airway epithelia cultures, primary HNE cells were infected with ethacridine-treated virus and incubated with the media containing 5 μM ethacridine for 48 hours (**Fig 5A**). Immunostaining with anti-N and anti-S antibodies in HNE cells showed that, in the presence of ethacridine, the proportion of infected primary nasal cells was dramatically decreased (**Fig 5B and 5C**), suggesting that ethacridine protected HNE cells from viral infection. These data suggest that the antiviral effect of ethacridine is cell type-independent, consistent with the main mode of action of this drug. Lastly, we examined whether ethacridine inhibits virus reproduction in the HNE cells by the same mechanism as in Vero E6 cells. We pre-incubated the virions with the drugs and then we added the mixtures to the HNE cells. Immediately after adsorption, we washed the sample and removed the drug. The cells were then incubated with drug-free medium. We conducted immunostaining at 48 hpi, which showed strong inhibition of the virions in the HNE cells (**S10 Fig**). Therefore, our data suggest that ethacridine inhibits the virions in the HNE cells by the same mechanism as in the Vero E6 cells.

## Discussion

Several small molecule-based inhibitors have been identified to interfere with major targets involved in the viral lifecycle of SARS-CoV-2, including the main protease Mpro and the SARS-CoV-2 replicase RdRp. For example, *α*-ketoamide inhibitors of Mpro and the FDA-approved drug ebselen have been reported to inhibit Mpro activity with EC50 at 0.53 and 4.67 *μ*M, respectively [8–10]. The replicase RdRp inhibitor, remdesivir, has been designed and approved by FDA to treat critically-ill COVID-19 patients [6,17]. However, very recent data from WHO showed no or little effect in hospitalized COVID-19 patients, including metrics of overall mortality, initiation of ventilation and duration of hospital stay [18]. Thus, therapeutic efficacy of remdesivir appears far from satisfactory, and new therapeutic options are urgently needed.

The current work started through development of a target-specific cell-based screening assay. We created the Mpro sensor, and our screen of 1622 FDA-approved drugs led to identification of 9 drugs that inhibit Mpro activity and show anti-SARS-CoV-2 activity. The new sensor adds to the pool of several reporters as powerful drug screening tools against the SARS-CoV-2 [19–21]. We characterized the antiviral property of five drugs further. Four of them, including simeprevir, ABT-199, hydroxyprogesterone and cinacalcet effectively inhibited Mpro and blocked SARS-CoV-2. This suggests that these latter four drugs inhibit SARS-CoV-2 mainly via inhibition of Mpro activity. These drugs have similar or better EC50 than ebselen, and thus they may be repurposed as potential therapies against COVID-19. Furthermore, previous computational docking predicted that simeprevir and venetoclax (ABT-199) bind and inhibit Mpro [22,23]. Hydroxyprogesterone was independently shown inhibits SARS-CoV-2, but its mode of action is unclear [24]. Our data suggests that hydroxyprogesterone may block SARS-CoV-2 by inhibiting Mpro activity. Utilizing our FlipGFP-based HTS approach enables screening of Mpro inhibitors in the cellular context, and can be expanded to visualize activity of key proteases of other viruses at BSL2, and to identify their inhibitors. Our HTS approach is complemental to other HTS-based methods, which together are useful technologies against SARS-CoV-2 [5,24–26].

The most potent antiviral drug discovered in the current work, ethacridine, showed higher antiviral potency than remdesivir and very little cell toxicity (**Fig 3E**). This agent blocks SARS-CoV-2 mainly by inactivating the viral particles. First, unlike the other identified drugs that show similar range of IC_50_ in inhibiting Mpro and EC50 against SARS-CoV-2, ethacridine has much higher anti-viral potency (EC_50_ ~ 0.08 μM) than its Mpro-inhibiting activity (IC_50_ ~ 3.5 *μ*M), indicating that the main mode of action of ethacridine is not via inhibition of Mpro. Second, we provide direct evidence that after ethacridine treatment, SARS-CoV-2 particles have markedly reduced ability to bind to host cells. On the other hand, ethacridine does not appear to block viral RNA replication as shown by the qRT-PCR data. Therefore, our data suggest that ethacridine blocks SARS-CoV-2 mainly by inactivating the viral particles with little effect on viral RNA replication. One possibility is that while ethacridine inactivates large amounts of virions, some virions may escape and these escaped virions would replicate in the cells, producing viral RNA. On the other hand, when the newly assembled virions are released from the cells into supernatant, they will be inactivated by the drug, resulting in low plaque number, but high viral RNAs. The precise mechanism for how ethacridine inactivates the viral particles and their infection will require further investigation, such as potential ultrastructural changes of viral particles and/or binding of ethacridine to viral RNA and/or proteins.

We also noticed that while ethacridine inhibits Mpro activity with IC50 = 3.54 μM, it has little effect on inhibiting the viral replication at 5 μM (see qRT-PCR of “DMSO + Eth.” on **Fig 4A**). This is likely due to discrepancy of an inhibitor on inhibiting Mpro activity (IC_50_) versus inhibiting viral replication (EC_50_). Such discrepancy between IC_50_ (metrics for inhibitor’s capability on Mpro activity inhibition) and EC_50_ (metrics for inhibitor’s capability on blocking virus replication) has been reported previously [8,10]. For example, ebselen was reported to have IC_50_ ~ 0.67 μM, but its EC_50_ is ~ 4.67 μM [8]. Other examples include two other Mpro inhibitors, compound 11a and 11b, which were rationally designed based on the Mpro structure. The IC_50_ for 11a and 11b was reported to be 53 and 40 nM; but their EC_50_ was 0.53 and 0.72 μM [10].

Our findings herein reveal a new approach against SARS-CoV-2 through the inactivation of viral particles, for which the efficacy is expected to be cell type-independent. Indeed, ethacridine blocks SARS-CoV-2 infection of human cell line A549^ACE2^ cells and, importantly, in primary human nasal epithelial cells. Because ethacridine has been safely used as a topical drug, one application is as a prophylactic agent to block SARS-CoV-2 infection. For example, ethacridine has been used in postoperative nasal infusion-irrigation for disinfection, after sinusotomy in patients with fungal infection of the sinuses [27]. Thus, ethacridine may be applied to nasal epithelia as a prophylactic treatment to prevent SARS-CoV-2 infection.

Ethacridine is highly soluble and non-toxic in various animal models including rat, mice and rabbits. For example, mice treated with 20 mg/kg ethacridine by i.p. injection showed no toxicity [28]. Ethacridine has also been administered into patients for treating puerperal sepsis via intravenous injection [29]. Ethacridine (also known as rivanol) has been used in the oral treatment of enteric diseases such as traveller’s diarrhoea and shigellosis. Thus, it is plausible to validate its antiviral effect in animal models and COVID-19 patients.

## Methods

### Plasmid construction

Plasmid constructs were created by standard molecular biology techniques and confirmed by exhaustively sequencing the cloned fragments. To create pcDNA3-Mpro-flipGFP-T2A-mCherry, the GFP10-E5-GFP11-Mprosubstrate-K5 fragment was first generated by PCR with an overlapping primer with coding sequence of the MPro substrate, AVLQSGFR. This fragment was then digested with NheI and AflII and inserted into the pcDNA3-TEV-flipGFP-T2A-mCherry [11].

### Characterization of GFP-based Mpro activity sensor FlipGFP^Mpro^

To check the FlipGFP^Mpro^ signal in the presence of wildtype or mutant MPro of SARS-CoV-2, HEK293T cells were seeded onto eight-well plate and 24 hours later, 40 ng of pcDNA3-FlipGFP^Mpro^-T2A-mCherry was transfected with 40 ng of pLVX-EF1alpha-nCoV2019-nsp5-2xStrep-IRES-Puro (expressing wildtype Mpro of SARS-CoV-2) or pLVX-EF1alpha-nCoV2019-nsp5-C145A-2xStrep-IRES-Puro (expressing C145A MPro mutant). To check the FlipGFP^Mpro^ signal after SARS-CoV-2 infection, HEK293T on 10-mm coverglasses were transfected with 200 ng of pcDNA3.1-hACE2 (Addgene, #145033) 24 hours later. Those 293T cells were further infected with SARS-CoV-2 at an MOI of 0.5. At 24 hours after infection, 293T cells were fixed and processed for immunostaining against dsRNA (Scicons, 10010500) and visualized with goat anti-mouse IgG H&L (Cy5) (Abcam, ab6563). Images were taken using Nikon Eclipse Ti-E Spinning Disk under 20X and 60X. The cell pixel intensity in the GFP and mCherry channels were scored using Analyze Particle function in ImageJ. The ratio of GFP pixel intensity against mCherry pixel intensity was then compared in cells with wildtype and mutant MPro or in cells with SARS-CoV-2 or mock infection.

### High-throughput screen (HTS) against the FDA-approved library

An FDA-approved drug library (MedChemExpress) containing 1622 compounds was used for HTS. For initial screening, 293T cells were seeded onto a 96-well plate, incubated overnight and transfected with 20 ng of pcDNA3-Mpro-flipGFP-T2A-mCherry and 20ng pLVX-EF1alpha-nCoV2019-nsp5-2xStrep-IRES-Puro encoding Mpro using calcium-phosphate transfection. At 3 hours after transfection, compounds were added to individual wells at a 100X dilution for a final concentration of 20 μM. Verification of the 120 hits from the initial screening and further IC_50_ testing of the 12 hits followed a similar protocol except that a decreasing concentration series was used. Images were acquired 20 hours after transfection in the GFP and mCherry channels with a Nikon Eclipse Ti-E Spinning Disk. The cells’ pixel intensity in the GFP and mCherry channels were scored using Analyze Particle function in imageJ. Mpro activity was calculated as the ratio of the GFP pixel intensity versus the mCherry pixel intensity.

### Antiviral assay

The African green monkey kidney Vero E6 cell line was obtained from the American Type Culture Collection (ATCC, no. 1586) and maintained in Minimum Essential Medium (MEM; Gibco Invitrogen) supplemented with 10% fetal bovine serum (FBS; Gibco Invitrogen), 1% penicillin-streptomycin-glutamine (Gibco Invitrogen), at 37°C in a humidified 5% CO_2_ incubator. A clinical isolate of SARS-CoV-2 (USA-WA1/2020, BEI Cat No: NR-52281) was propagated in Vero E6 cells. Viral titers were quantified with a plaque assay. All the infections were performed at biosafety level-3 (BSL-3).

To assess the antiviral activity, ~70% confluent monolayers of Vero E6 cells (3×10^5^ cells/well in 24-well plates) were pretreated with drugs at different concentration for 3 hours (pretreatment) and then infected with SARS-CoV-2 (MOI = 0.5) at 37°C for 1 hour. The virus solution was removed, cells were further cultured with fresh medium containing drugs at different concentrations. At 16 hours post-infection, viral titers of the supernatants were detected with a plaque assay.

### Plaque assay

Confluent monolayers of Vero E6 cells grown in six-well plates were incubated with the serial dilutions of virus samples (250 μl/well) at 37°C for 1 hour. Next, the cells were overlayed with 1% agarose (Invitrogen) prepared with MEM supplemented with 2% FBS. Three days later, cells were fixed with 4% formaldehyde for 2 hours, the overlay was discarded, and samples were stained with crystal violet dye.

### Viral RNA quantification

Viral RNA (vRNA) was extracted from cell pellets or supernatants with Tri-reagent (Ambion), following the manufacturer’s instructions. The supernatant samples were pretreated with RNAse A for 2 hours at 37°C to remove non-encapsidated RNA. RNA from supernatants was used directly to make cDNA using the iScript RT Supermix (BioRad). For cell pellets, 1–2 mg of RNA was treated with DNAse I (NEB), 10% of this reaction was used to make cDNA. qPCR was done using the Luna Universal qPCR Master Mix (NEB) and run on a CFX connect qPCR Detection System (BioRad). To determine the number of vRNA copies per ml, plasmids containing the nucleocapsid gene of SARS-CoV-2 (cloned from the USA-WA1/2020 isolate) were used as standards and diluted serially 10-fold to determine target copy numbers. Threshold cycle (Ct) values were plotted against the number of target-copies, and the resultant standard curve was used to determine the number of genome equivalents of vRNA in the samples. For cell pellet samples, the vRNA copy number was normalized to the housekeeping gene *huel* [30]. All samples were within the range of linearity of a standard curve and primers efficiencies were 100% +/- 5%. The primers used for SARS-CoV-2 are 5′-TCCTGGTGATTCTTCTTCAGG-3′ and 5′-TCTGAGAGAGGGTCAAGTGC-3′ and for huel 5′-TCAGACGACGAAGTCCCCATGAAG-3′ and 5′-TCCTTACGCAATTTTTTCTCTCTGGC-3′

### Cytotoxicity assay

Cytotoxicity of the identified drugs on Vero E6 cell was determined with WST-1 cell proliferation assays (ROCHE, 5015944001). Twenty thousand cells were seeded into a 96-well plate and incubated for 20–24 h at 37°C, and 1 μL of each compound at decreasing concentrations was added. After 18 hrs incubation at 37°C, WST-1 assays were performed according to manufacturer’s protocols. All experiments were performed in triplicate.

### Adsorption assay

The virus sample was supplemented with 5 μM ethacridine or with DMSO and incubated for 1 hour at 37°C. Samples were added to ~70% Vero E6 cells monolayers on coverslips with an MOI of 100. The cells were immediately placed on ice for 1 hour, and then the virus suspension was quickly removed, and cells were washed three times with ice-cold PBS. Cells were immediately fixed with 4% PFA for 30 min at room temperature. PFA was further washed with PBS and quenched with 1 M glycine in PBS. Immunostaining was further carried out using an antibody against nucleocapsid (Genetex, SARS-CoV-2 (COVID-19) nucleocapsid antibody, GTX135357) or an antibody against spike (Genetex, SARS-CoV-2 (COVID-19) spike antibody [1A9], GTX632604) and visualized with goat anti-rabbit IgG H&L (Alexa Fluor488) (Abcam, ab150077) or goat anti-mouse IgG H&L (Alexa Fluor555) (Abcam, ab150114). Images were acquired using Nikon Eclipse Ti-E Spinning Disk under 60X and processed in Image J. Fluorescence signals were calculated by pixel intensity using Analyze Particle function in Image J.

### Viral infectivity assay after ethacridine incubation

Virus sample (3X10^6^ pfu/ml) was supplemented with 5μM ethacridine or with DMSO and incubated for 1 hour at 37°C. The mix were then diluted around for 3.8X10^4^ fold and 0.25ml of the dilution was added to plate with Vero E6 cells or A549^ACE2^ cells. After 1-hour incubation under 37°C, 3 ml agar was overlaid for plaque assay.

### Air-liquid interface culture of HNEs

Primary human nasal epithelial cells (HNE), from PromoCell, were cultured in suspension in PneumaCult Plus Medium according to manufacturer instructions (StemCell Technologies, Cambridge, MA, USA). To generate air-liquid interface cultures, HNE cells were plated on 0.4 μm pore polyester membrane inserts with a 0.4-micron pore size (Corning, Tewksbury, MA, USA) at 3.3 x 10^4^ cells in 0.2 mL PneumaCult-Ex Plus Medium and inserted into 24 well culture plates. Cells were maintained for the first 2~4 days in PneumaCult Plus Medium, until confluence is reached. Medium were gently aspirated from both the basal and apical chambers, then 0.5 ml PneumaCult-ALI Maintenance Medium was added to the basal chamber only. Cells were incubated at 37°C and full medium change in the basal chamber with PneumaCult-ALI Maintenance Medium was performed every 2 days. HNE cells were maintained at air-liquid interface for 28 days for differentiation.

### Virus vs Cell treatment with ethacridine for virus inactivation

To discriminate if ethacridine targets SARS-CoV-2 particles or some cellular components, cells and viruses were treated separately before infection. For treating cells, confluent monolayers of Vero E6 grown in six-well plates were incubated with DMSO or 5uM ethacridine at 37°C for 3 hours and washed with PBS for 3 times. Next, cells were exposed to the serial dilutions of virus preparations (250 μl/well) for 1 hour at 37°C. Cells were overlayed with 1% agarose (Invitrogen) prepared with MEM supplemented with 2% FBS and processed as a regular plaque assay.

For virus treatment, sample (3X10^6^ pfu/ml) was supplemented with 5μM ethacridine or DMSO and incubated for 3 hours at 37°C. Serial dilutions of the resulting mixtures (0.25ml) were added to plates with Vero E6 cells, incubated for 1 hour at 37°C, and processed as a regular plaque assay.

### Validation of antiviral activity in HNEs

A clinical isolate of SARS-CoV-2 amplified in A549 cells overexpressing ACE2 was used. For HNE cell infected with high titer virus (MOI = 20), HNE cells were overlayed apically with 100 ul of the prepared virus media and incubated for 1 hour at 4C. After washing 3 times with ice-cold PBS, the cells were fixed. For validating antiviral activity of ethacridine in HNEs, virus samples were pretreated with 5 μM ethacridine or with DMSO for 1 h at 37°C. HNE cells were overlayed apically with 100 ul of the resulting media (MOI = 5 or 0.1) and incubated for 1 hour at 4°C. Cells were washed 3 times with ice-cold PBS, the liquid from the apical chamber was aspirated, and the transwells were incubated in the fresh PneumaCult-ALI Medium in the presence or absence of 5 μM ethacridine. 48 hours after infection, cells were fixed with 4% PFA for 30 min, washed once with PBS, quenched with 1M glycine/PBS, and proceeded for immunostaining.

### HNE whole-mount immunostaining and imaging

Fixed SARS-CoV-2-infected HNE ALI cultures were fixed for 30 min in 4% formaldehyde in PBS, washed and stored in PBS. Filters were excised from plastic supports. After blocking with 5% normal donkey serum (017-000-121, Jackson ImmunoResearch) in immunofluorescence (IF) buffer (3% BSA and 0.1% NP-40 in PBS) for 1 hour at room temperature, cells were incubated with primary antibody in IF buffer for overnight at 4°C, followed by rinsing with IF buffer five times. The samples were then incubated with fluorescent-labeled secondary antibody and phalloidin (A30107, Invitrogen) in IF buffer for 1 hour at room temperature, followed by rinsing with IF buffer five times. After nuclear staining with 4, 6-diamidino-2-phenylindole (DAPI) in PBS, filters were mounted with Fluoromount-G (0100–01, SouthernBiotech) onto glass slides. The samples were visualized using a LSM880 Airyscan system (Carl Zeiss) with a Plan ApochromatX60 objective. Infection ratio was calculated as the (number of SPIKE or nucleocapsid protein positive cells)/(total cell number counted with DAPI). Cell count function in Image J was used for cell counting.

### Statistics

All statistics were performed in GraphPad Prism. IC_50_ or EC_50_ of MPro inhibition, cytotoxicity and antiviral activity was calculated using the non-linear fit function (Variable slope). Non-paired t-test was used to compare differences between groups. One way-ANOVA and Dunnett’s multiple comparisons test was used to compare differences among multiple groups.

## Supporting information

S1 Fig Verification of the selected top 120 drugs using the Mpro activity reporter FlipGFP^Mpro^. Ratio of Mpro activity was calculated based on FlipGFP^Mpro^ fluorescence of drug-incubated HEK293 cells, divided by that of DMSO-treated HEK293 cells. FlipGFP^Mpro^ fluorescence was normalized by mCherry in HEK293 cells, which co-expressed FlipGFP^Mpro^, mCherry, and Mpro. Data are mean ± SD (n = 5).
(TIF)

S2 Fig Dose-response curve of Mpro inhibition. The Mpro activity was determined as FlipGFP fluorescence normalized by mCherry. The ratios of Mpro activity were calculated by normalizing Mpro activity with that of cells treated with DMSO. Inhibition ratio was calculated as (1-(ratio of Mpro activity)) X100%. Data are mean ± SD (n = 5). IC_50_ was represented as mean ± SEM (n = 5).
(TIF)

S3 Fig Cytotoxicity of the identified drugs in HEK293T cells. Cell-toxicity curve of each drug against SARS-CoV-2. CC_50_ is represented as mean ± SEM (n = 3).
(TIF)

S4 Fig Antiviral activities of the identified drugs. Antiviral activities of five drugs (5 *μ*M) were quantified by a plaque assay with SARS-CoV-2 in Vero E6 cells. Data are mean ± SD (n = 3). *: p value < 0.05; ***: p value < 0.001. ns: not significant. PFU: plaque-forming unit.
(TIF)

S5 Fig Cytotoxicity of the identified drugs in Vero cells. The cytotoxicities of the indicated compounds were determined in Vero E6 cells with the WST-1 assay. CC_50_ is represented as mean ± SEM (n = 3).
(TIF)

S6 Fig Antiviral effects of ethacridine on SARS-CoV-2 by plaque assays. 1) cell+DMSO: Vero cells were treated with DMSO for 3 hours. The sample was washed, immediately prior to addition of SARS-CoV-2 virus, followed by plaque assay; 2) cell+eth: Vero cells were treated with 5uM ethacridine for 3 hours. The sample was washed, immediately prior to addition of SARS-CoV-2 virus, followed by plaque assay; 3) virus+DMSO: SARS-CoV-2 was treated with DMSO for 3 hours. The mixture was added to Vero cells for plaque assay; 4) virus+eth: SARS-CoV-2 was treated with 5 *μ*M ethacridine for 3 hours. The mixture was added to Vero cells for plaque assay (the drug was significantly diluted); The data are shown as mean +/- SEM (n = 3). P values are calculated to be: 1) cell+dmso vs cell+eth, *p = 0*.*398*. 2) virus+dmso vs virus+eth, *p = 0*.*001*.
(TIF)

S7 Fig Small antiviral effect on SARS-CoV-2 with no pretreatment of ethacridine with the virus. The virus was mixed (no pretreatment) with DMSO or ethacridine (5 μM), and the mixture was immediately added to Vero E6 cell for adsorption (1 h at 37°C). Then agarized media was added for plaque assay. It showed ~2.65 fold inhibition (P = 0.004). This inhibitory effect is ~1000 times smaller than that with drug pretreatment with the virus (Fig 4A, S6 Fig).
(TIF)

S8 Fig Virucide effect of ethacridine on SARS-CoV-2. Effects of ethacridine on the infectivity of SARS-CoV-2 were examined using plaque assay at 37°C (**a**, **b**) or in the room temperature (RT) (**c**, **d**). SARS-CoV-2 was mixed with ethacridine for 1 or 2 hours before being added to infect Vero E6 cells. Data are mean ± SEM (n = 3).
(TIF)

S9 Fig Quantitative analysis of viral titer by plaque assay in the human cells A549 that stably express ACE2. SARS-CoV-2 was pre-incubated with ethacridine (5 uM) for 1hr, followed by plaque assay on the human A549 cells stably expressing human ACE2 (A549^ACE2^).
(TIF)

S10 Fig Antiviral effects of ethacridine in the HNE cells. SARS-CoV-2. (MOI = 0.1) was incubated with DMSO or 5 *μ*M ethacridine for 1 hour. The mixture was then added to the HNE cells for adsorption at 4C for 1 hour. The sample was then washed and the drug was removed. The sample was added with drug-free fresh medium. The cells were fixed 48 hpi, followed by immunofluorescence imaging. The data are shown as mean +/- SEM (n = 3). The quantitative analysis was conducted by imaging 12 ROIs from 3 samples (infection ratio of the ethacridine-treated sample = 0, *p<0*.*0001)*.
(TIF)
