# Supplementary figures and images for: Ethacridine inhibits SARS-CoV-2 by inactivating viral particles
Source: PLoS Pathog. 2021 Sep 3;17(9):e1009898. doi: 10.1371/journal.ppat.1009898 (PMC8445489; doi:10.1371/journal.ppat.1009898)

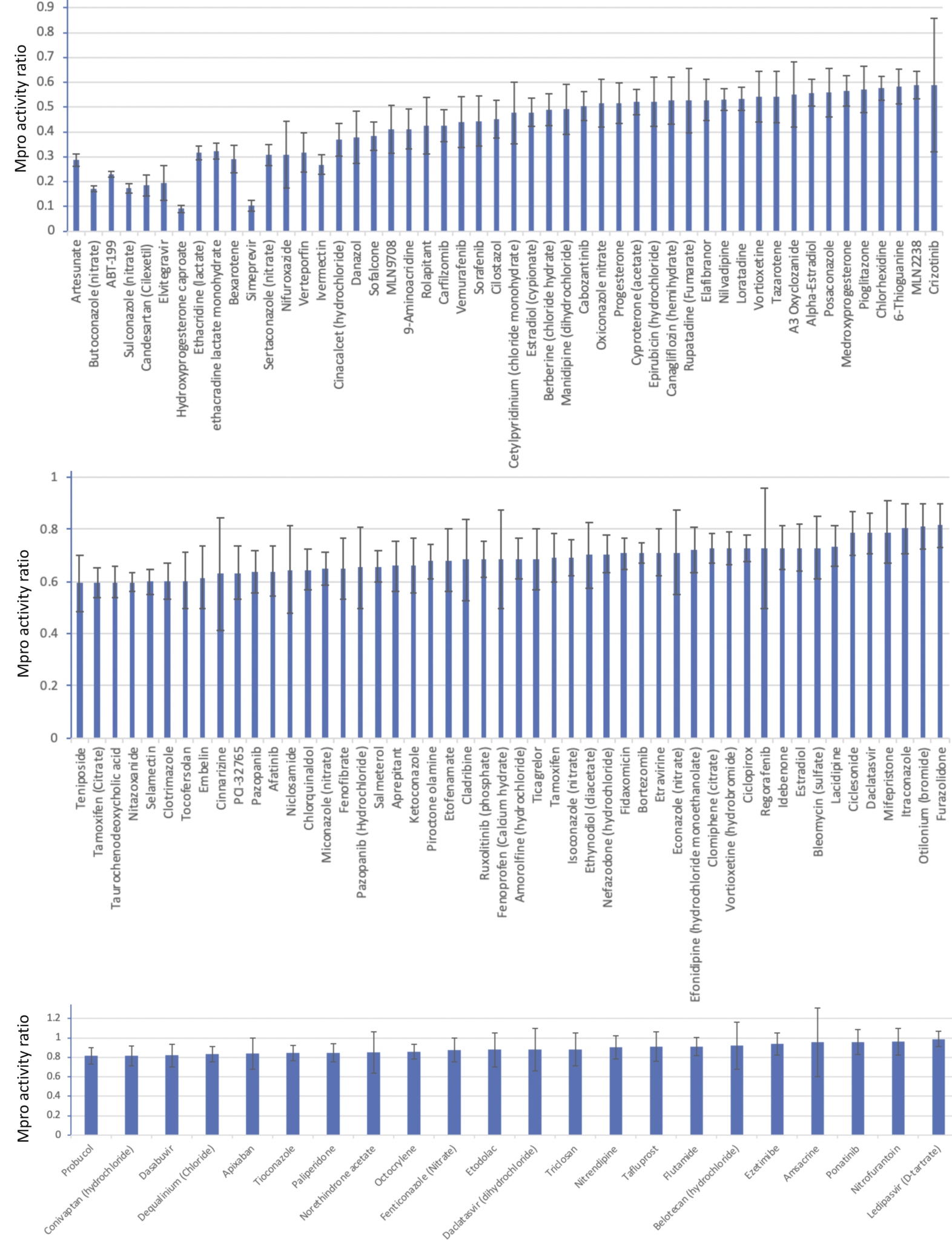

Supplement: S1 Fig — Ratio of Mpro activity was calculated based on FlipGFPMpro fluorescence of drug-incubated HEK293 cells, divided by that of DMSO-treated HEK293 cells. FlipGFPMpro fluorescence was normalized by mCherry in HEK293 cells, which co-expressed FlipGFPMpro, mCherry, and Mpro. Data are mean ± SD (n = 5). (TIF) [file ppat.1009898.s001.tif]

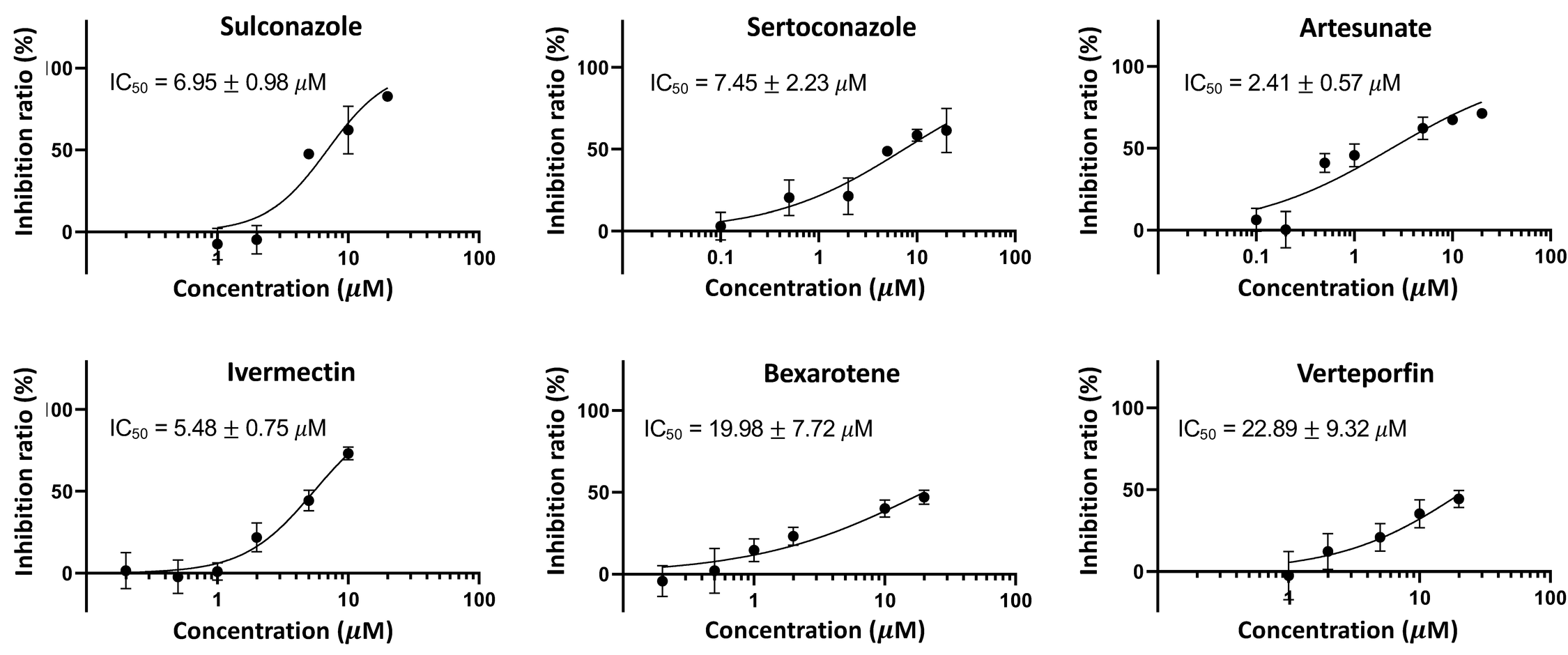

Supplement: S2 Fig — The Mpro activity was determined as FlipGFP fluorescence normalized by mCherry. The ratios of Mpro activity were calculated by normalizing Mpro activity with that of cells treated with DMSO. Inhibition ratio was calculated as (1-(ratio of Mpro activity)) X100%. Data are mean ± SD (n = 5). IC50 was represented as mean ± SEM (n = 5). (TIF) [file ppat.1009898.s002.tif]

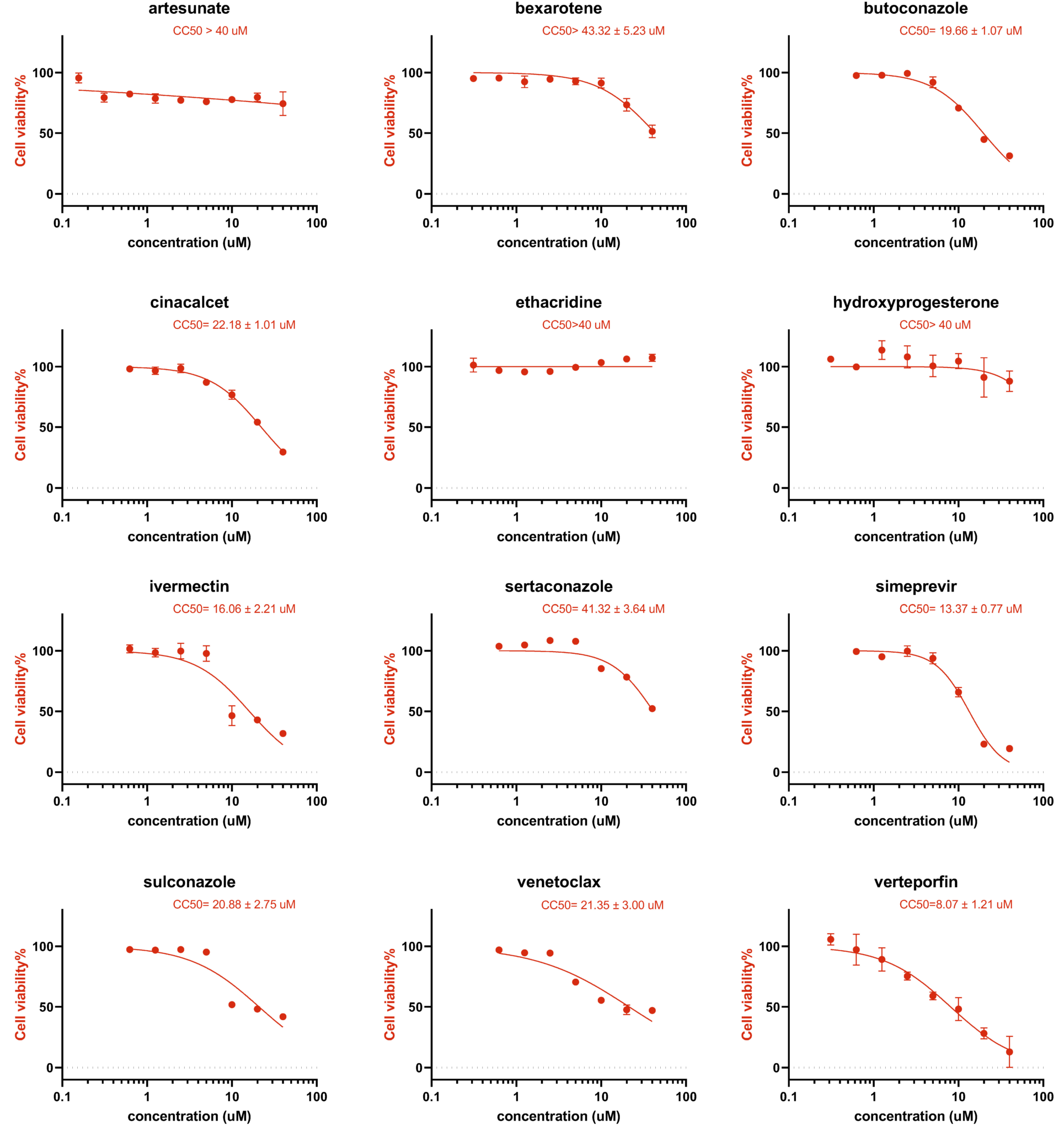

Supplement: S3 Fig — Cell-toxicity curve of each drug against SARS-CoV-2. CC50 is represented as mean ± SEM (n = 3). (TIF) [file ppat.1009898.s003.tif]

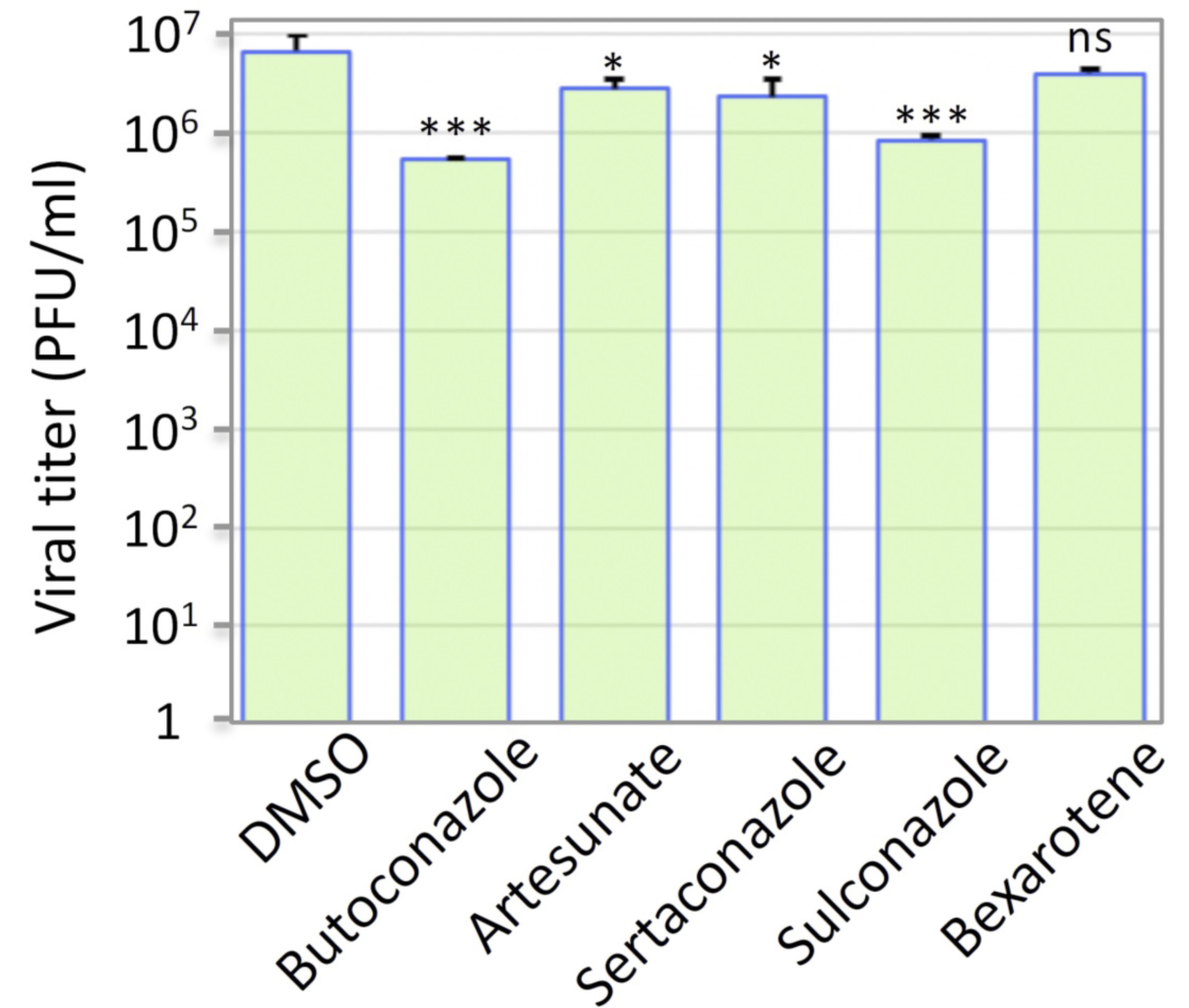

Supplement: S4 Fig — Antiviral activities of five drugs (5 μM) were quantified by a plaque assay with SARS-CoV-2 in Vero E6 cells. Data are mean ± SD (n = 3). *: p value < 0.05; ***: p value < 0.001. ns: not significant. PFU: plaque-forming unit. (TIF) [file ppat.1009898.s004.tif]

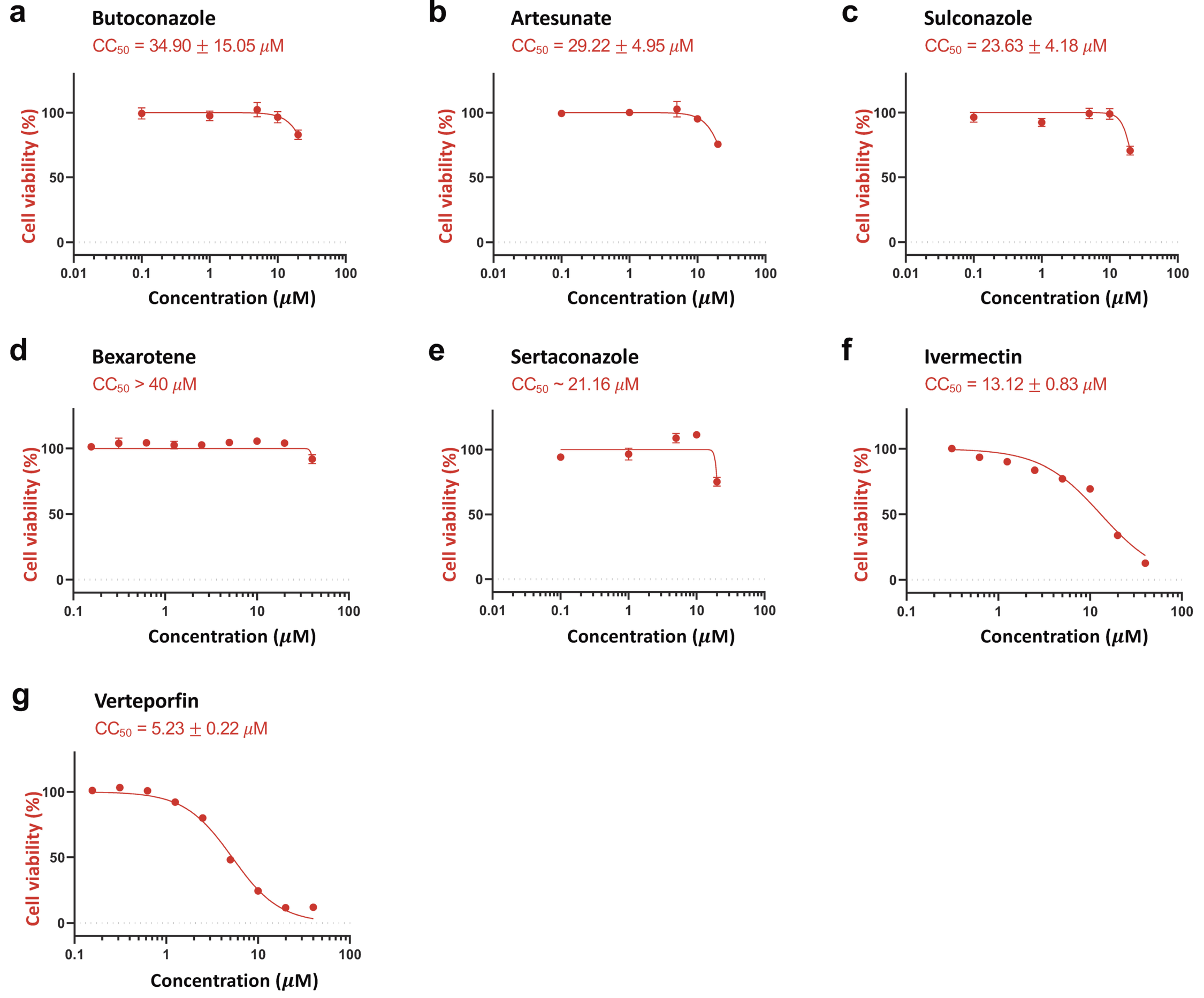

Supplement: S5 Fig — The cytotoxicities of the indicated compounds were determined in Vero E6 cells with the WST-1 assay. CC50 is represented as mean ± SEM (n = 3). (TIF) [file ppat.1009898.s005.tif]

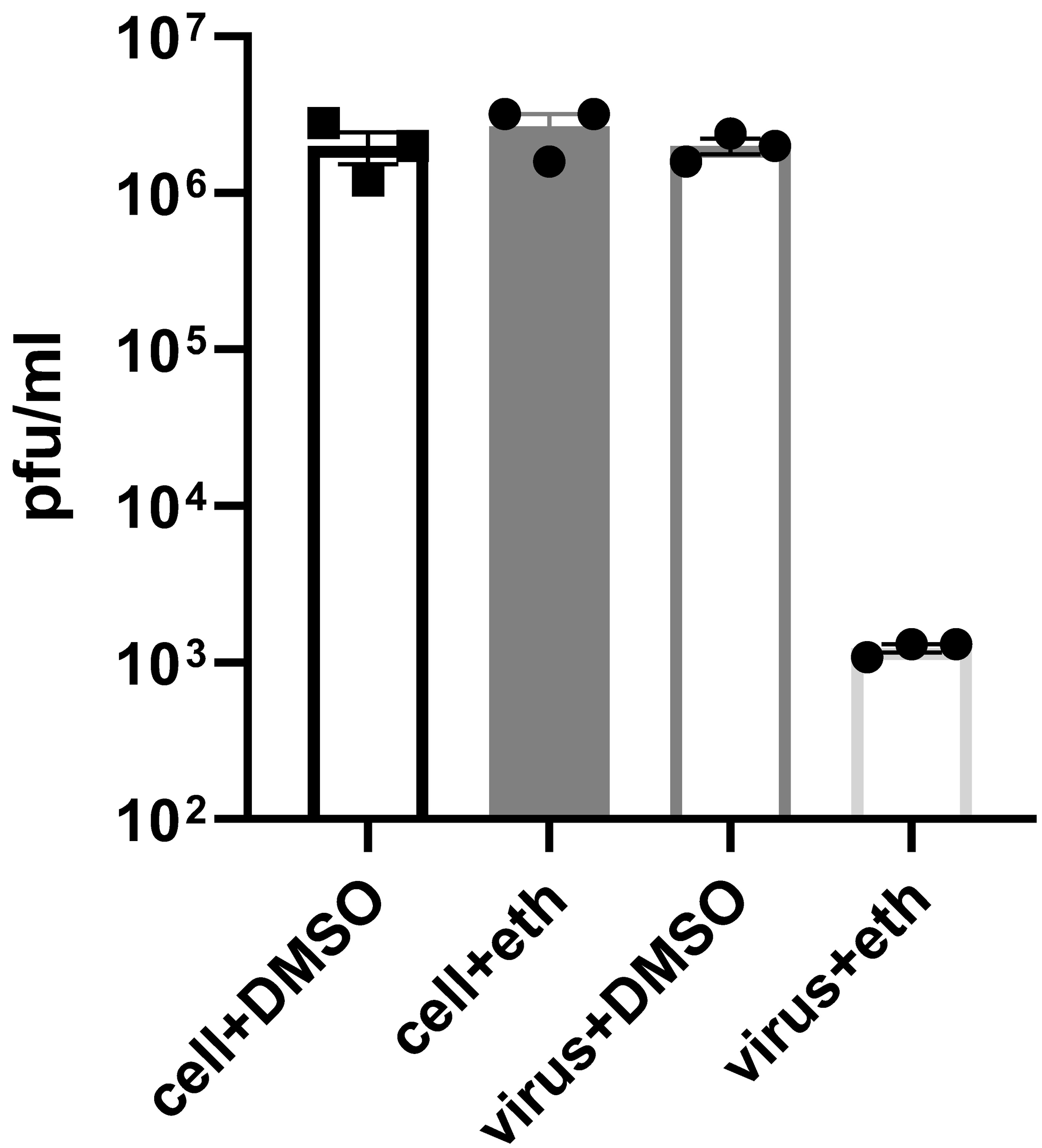

Supplement: S6 Fig — 1) cell+DMSO: Vero cells were treated with DMSO for 3 hours. The sample was washed, immediately prior to addition of SARS-CoV-2 virus, followed by plaque assay; 2) cell+eth: Vero cells were treated with 5uM ethacridine for 3 hours. The sample was washed, immediately prior to addition of SARS-CoV-2 virus, followed by plaque assay; 3) virus+DMSO: SARS-CoV-2 was treated with DMSO for 3 hours. The mixture was added to Vero cells for plaque assay; 4) virus+eth: SARS-CoV-2 was treated with 5 μM ethacridine for 3 hours. The mixture was added to Vero cells for plaque assay (the drug was significantly diluted); The data are shown as mean +/- SEM (n = 3). P values are calculated to be: 1) cell+dmso vs cell+eth, p = 0.398. 2) virus+dmso vs virus+eth, p = 0.001. (TIF) [file ppat.1009898.s006.tif]

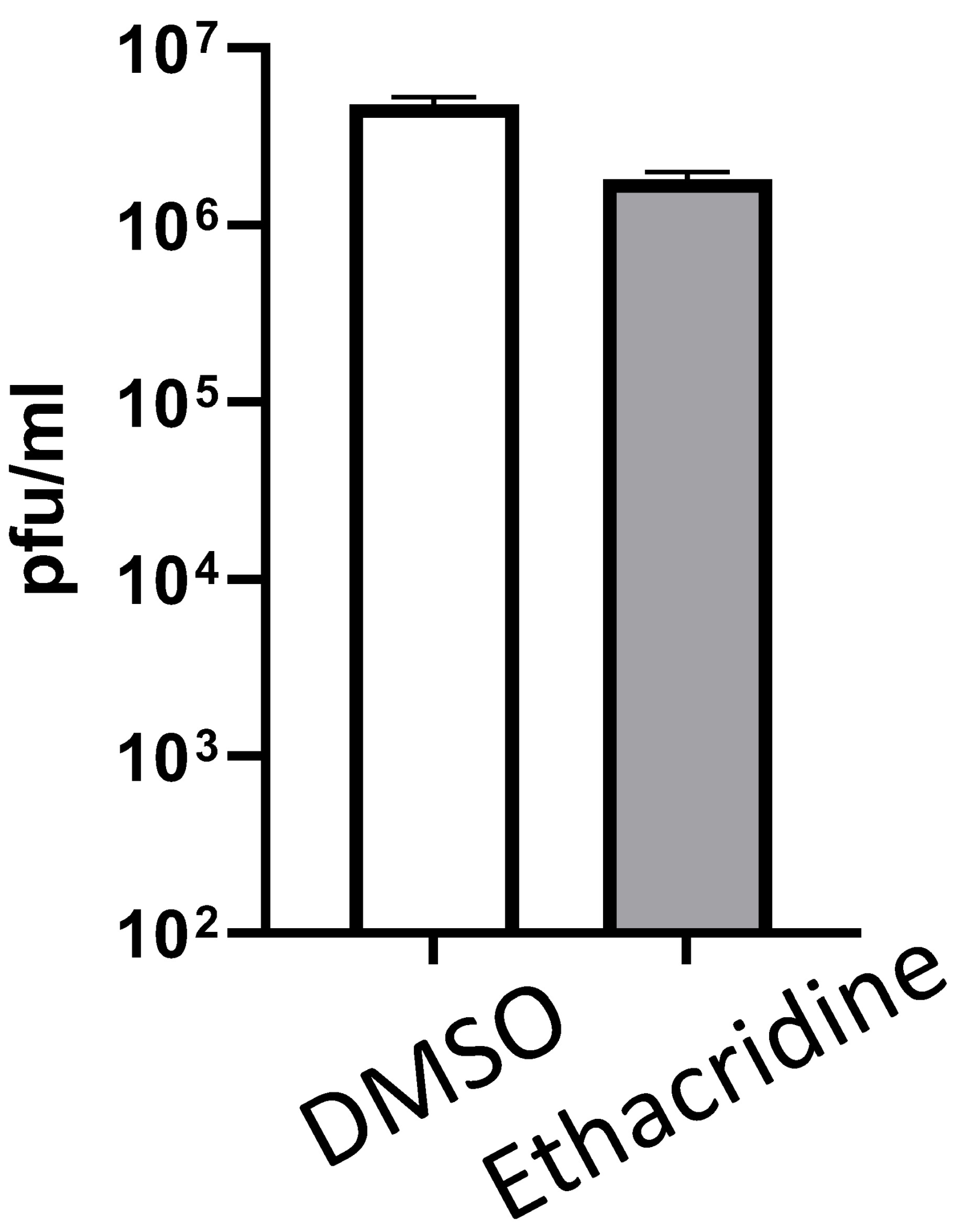

Supplement: S7 Fig — The virus was mixed (no pretreatment) with DMSO or ethacridine (5 μM), and the mixture was immediately added to Vero E6 cell for adsorption (1 h at 37°C). Then agarized media was added for plaque assay. It showed ~2.65 fold inhibition (P = 0.004). This inhibitory effect is ~1000 times smaller than that with drug pretreatment with the virus (Fig 4A, S6 Fig). (TIF) [file ppat.1009898.s007.tif]

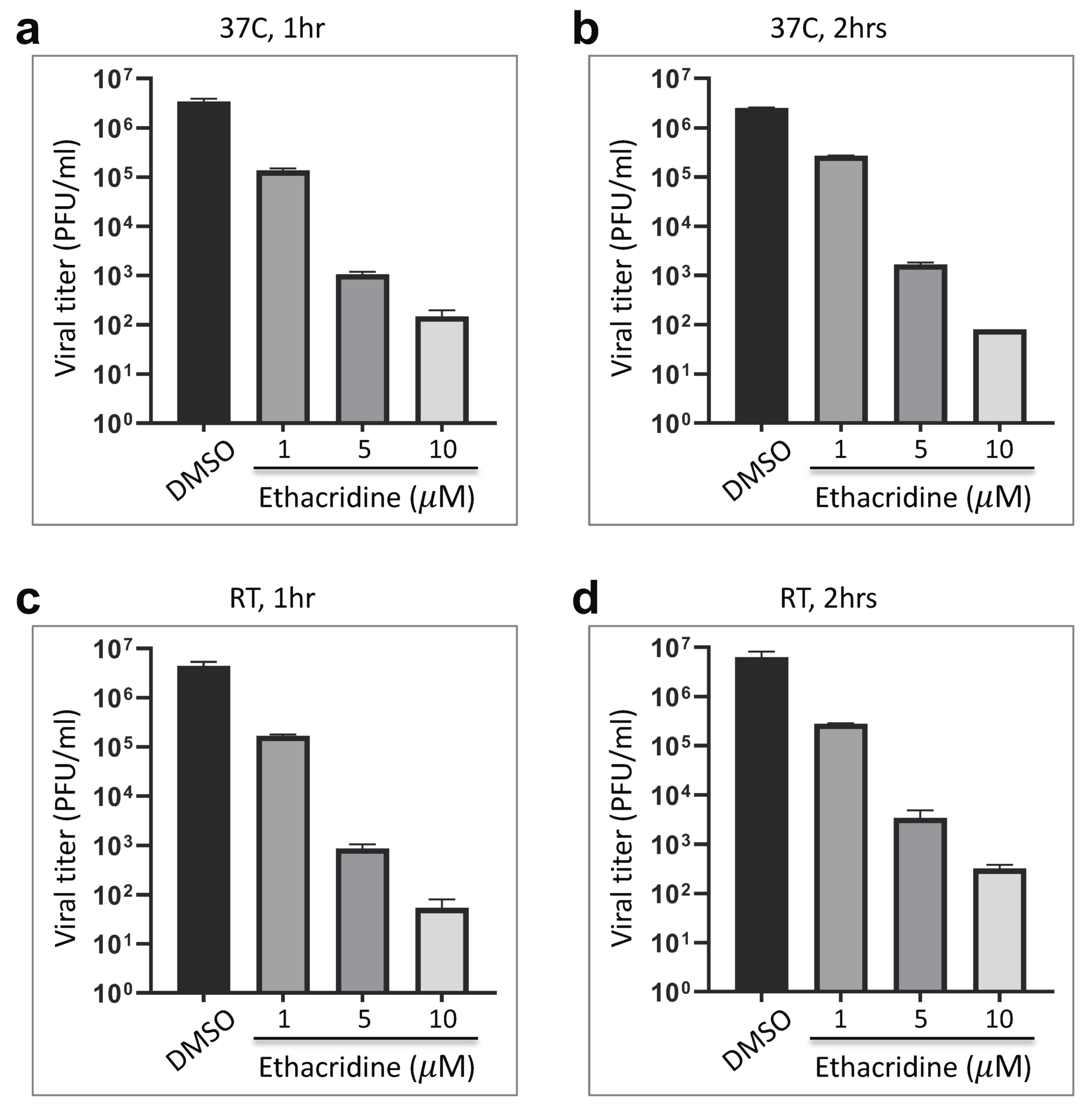

Supplement: S8 Fig — Effects of ethacridine on the infectivity of SARS-CoV-2 were examined using plaque assay at 37°C (a, b) or in the room temperature (RT) (c, d). SARS-CoV-2 was mixed with ethacridine for 1 or 2 hours before being added to infect Vero E6 cells. Data are mean ± SEM (n = 3). (TIF) [file ppat.1009898.s008.tif]

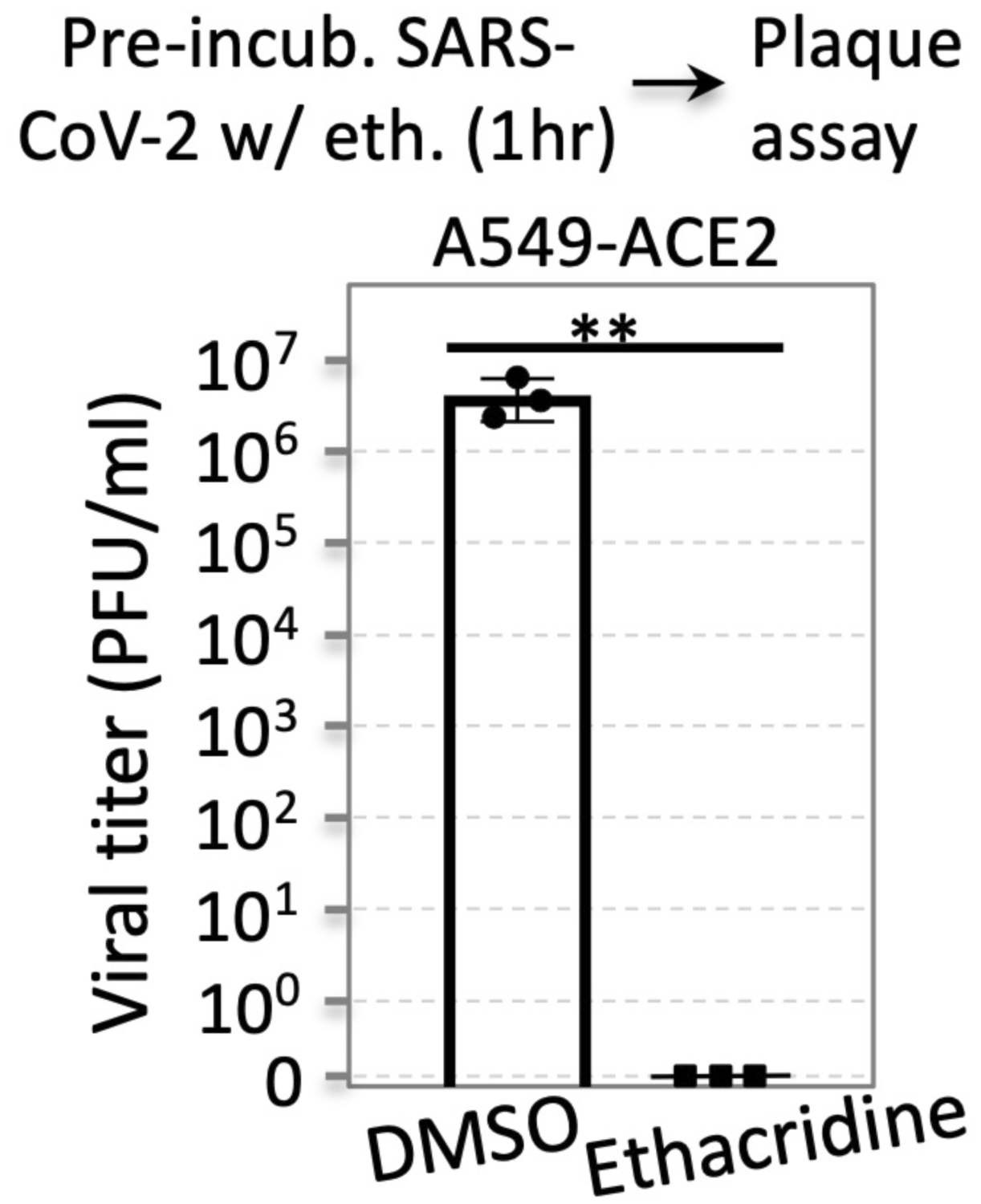

Supplement: S9 Fig — SARS-CoV-2 was pre-incubated with ethacridine (5 uM) for 1hr, followed by plaque assay on the human A549 cells stably expressing human ACE2 (A549ACE2). (TIF) [file ppat.1009898.s009.tif]

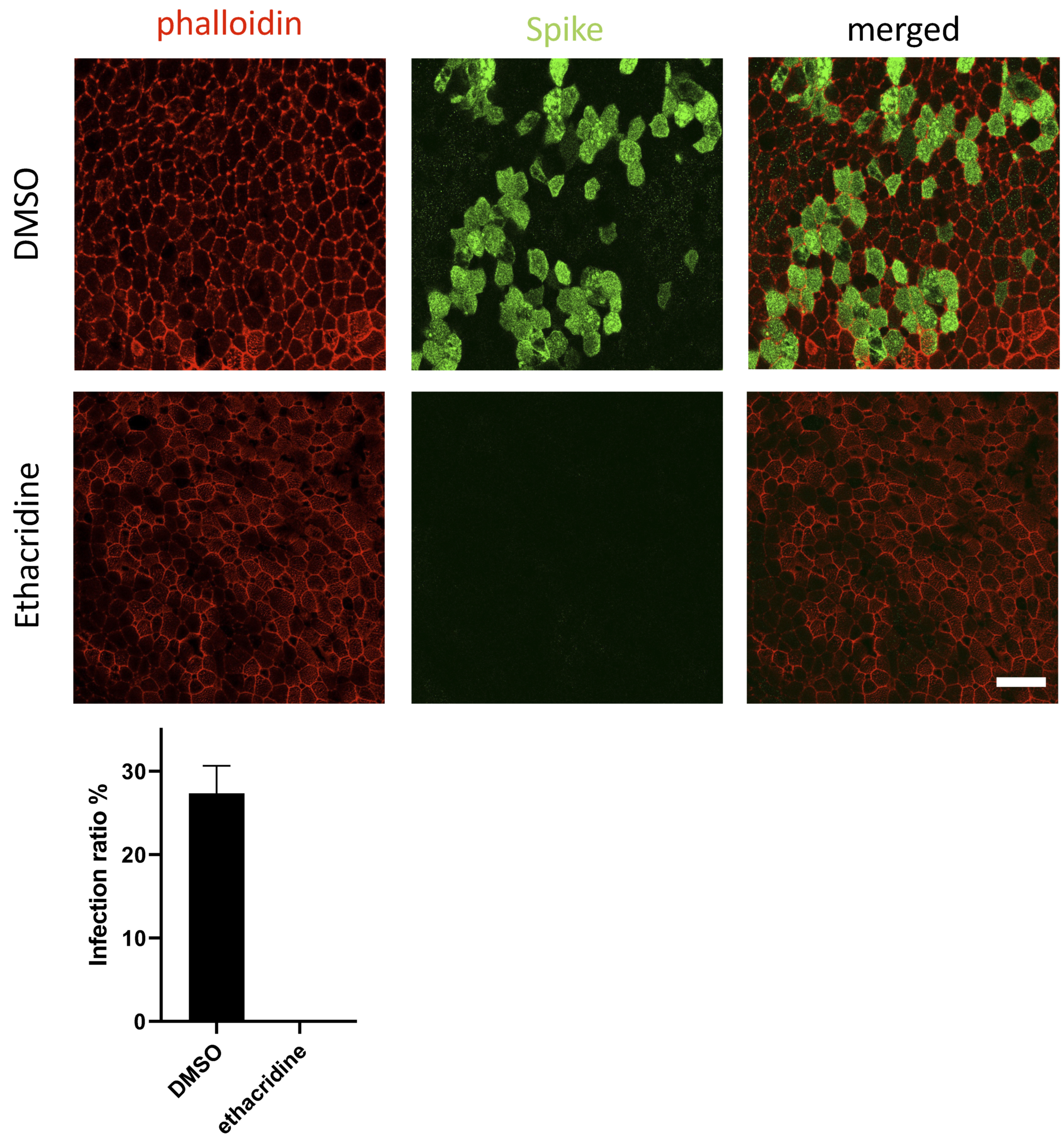

Supplement: S10 Fig — SARS-CoV-2. (MOI = 0.1) was incubated with DMSO or 5 μM ethacridine for 1 hour. The mixture was then added to the HNE cells for adsorption at 4C for 1 hour. The sample was then washed and the drug was removed. The sample was added with drug-free fresh medium. The cells were fixed 48 hpi, followed by immunofluorescence imaging. The data are shown as mean +/- SEM (n = 3). The quantitative analysis was conducted by imaging 12 ROIs from 3 samples (infection ratio of the ethacridine-treated sample = 0, p<0.0001). (TIF) [file ppat.1009898.s010.tif]
